# A Nutritional Supplement Containing Curcumin C3 Complex, Glucosamine, and Chondroitin Alleviates Osteoarthritis in Mice and Canines

**DOI:** 10.3390/vetsci12050462

**Published:** 2025-05-12

**Authors:** Enpei Zheng, Ting Cen, Ye Ma, Ziyuan Weng, Chuanheng Jiang, Luxi Hou, Jun Leng, Changmin Hu

**Affiliations:** 1 Department of Clinical Veterinary Medicine, College of Veterinary Medicine, Huazhong Agricultural University, Wuhan 430070, China; zep2023@webmail.hzau.edu.cn (E.Z.); cen15225336226@163.com (T.C.); mayeczj@webmail.hzau.edu.cn (Y.M.); guiqideyouxiang@163.com (Z.W.); 15680039817@163.com (C.J.); 2 Shenzhen Redray Biotechnology Co., Ltd., Shenzhen 518100, China; 15884401207@163.com (L.H.); lenjun@reddog.com.cn (J.L.); 3 The Veterinary Teaching Hospital, College of Veterinary Medicine, Huazhong Agricultural University, Wuhan 430070, China

**Keywords:** osteoarthritis, Curcumin C3 Complex, chondroprotection, anti-inflammatory, preclinical models

## Abstract

Osteoarthritis (OA) is a chronically progressive degenerative arthropathy characterized by the loss of cartilage, changes in subchondral architecture, and ongoing inflammation resulting in reduced mobility and pain. This study assessed the treatment potential of a combination of chondroitin and glucosamine enriched with Curcumin C3 Complex (C3GC) in modulating the pathophysiological features in mouse models with surgically induced OA and in dogs with naturally occurring OA. A cohort of 24 male C57BL/6 mice aged 3 months old were surgically destabilized with medial meniscus (DMM) to cause osteoarthritis. These animals underwent a nutritional intervention with C3GC or with GC over a course of 8 weeks. In order to evaluate cartilage health and subchondral bone structure, we carried out a combination of behavioral tests, micro-computed tomography (micro-CT), and histopathological examinations. In addition, a cohort of 12 OA-diagnosed retired police dogs were administered C3GC supplements or conventional care over a course of 30 days, with pain measurement and serum concentrations of MMP-3 and TNF-α determined before and after treatment. According to our findings, the administration of C3GC was determined to preserve subchondral microarchitectural structure integrity (*p* < 0.05) and resulted in better motor function in comparison with GC. In animals taking nutritional supplements, the OARSI scores of joint tissue sections were reduced, with the medial tibial plateau OARSI score being particularly low in the C3GC group (*p* < 0.0001). In dogs, treatment with C3GC resulted in a 24.5% reduction in serum MMP-3 levels (*p* < 0.01), and there was also a 20.8% decrease in serum TNF-α levels (*p* < 0.05), along with a decrease in subjective pain assessment. The results are in support of the chondroprotective, anti-inflammatory, and analgesic properties of C3GC and justify future research on the potential utility of C3GC in treating osteoarthritis.

## Simple Summary

This study evaluates the use of Curcumin C3 Complex-glucosamine-chondroitin (C3GC) to alleviate osteoarthritis (OA) symptoms in dogs and mice. OA was induced in mice through medial meniscus destabilization, and the subjects received 8 weeks of dietary supplementation with glucosamine-chondroitin (GC) alone or in combination with Curcumin C3 Complex (C3C). The findings showed that C3GC offers greater bone and cartilage protection than the use of GC alone. Supplementation in OA canines significantly decreased pain scores, serum MMP-3, and TNF-α, reflecting anti-inflammatory activity. The findings support the use of C3GC for alleviating OA in mice and dogs, though further clinical evidence is needed.

## 1. Introduction

Osteoarthritis (OA) is a common disease in older dogs, and the pain and inflammation it causes can affect the quality of life of the animals. Joint instability, incoordination, uneven weight bearing, and damage caused by developmental disorders, trauma, or iatrogenic factors can lead to abnormal pressure on the articular cartilage and chronic inflammation, thereby inducing osteoarthritis in animals [1].

Different studies have reported varying incidence rates of canine OA. In the UK, the incidence rate reported in primary veterinary clinics is 6.6%, while statistics from referral hospitals indicate 20%. Referral data in North America suggest that the incidence of OA in dogs over 1 year old is 20% and 80% in dogs over 8 years old. Overall, the incidence increases with age, and the commonly affected joints in animals are the knee joint, hip joint, and elbow joint [2]. Additionally, osteoarthritis in pet dogs can impose economic and psychological burdens on their owners. In 2003, the expenditure on cruciate ligament diseases in dogs alone in the United States was USD 1.32 billion [3].

The treatment options for osteoarthritis are relatively scarce; moreover, non-surgical therapies typically only alleviate symptoms, while surgical methods are considered a last resort. Clinical non-surgical methods primarily involve multimodal therapies, including pharmacological pain relief, non-pharmacological pain relief, appropriate exercise, weight management, and supplementation with joint nutrients. Although non-steroidal anti-inflammatory drugs (NSAIDs) have a beneficial effect on alleviating osteoarthritis, the risk of organ damage from long-term use of NSAIDs cannot be ignored [4]. Curcumin has demonstrated chondroprotective activity through the inhibition of NF-κB signaling, inhibition of matrix metalloproteinases (MMPs), and decreased oxidative stress in models of osteoarthritis [5]. Studies of human osteoarthritis patients have demonstrated a decrease in pain scores similar to those achieved with non-steroidal anti-inflammatory drugs (NSAIDs) when curcumin is administered in doses of more than 500 mg daily [6]. Its bioavailability continues to be a significant issue, and hence formulations that increase absorption, such as C3GC, are required. The present study evaluates the therapeutic efficacy of C3GC in osteoarthritic police dogs, a population with excessive joint loading due to physical exercise. Current clinical research extensively investigates curcumin’s therapeutic potential in osteoarthritis treatment and nutritional supplementation [7]. Mechanistic studies have progressively elucidated its key pharmacological actions, particularly focusing on anti-inflammatory and antioxidant properties. These investigations further demonstrate curcumin’s chondroprotective effects through both cellular preservation and apoptosis inhibition in chondrocytes [8]. Additionally, curcumin has shown therapeutic or delaying effects on diseases such as Alzheimer’s disease, rheumatoid arthritis, liver diseases, and diabetes in related studies involving humans or rats. In dogs, they have demonstrated effects in the treatment of osteoarthritis and degenerative myelopathy, as well as liver regeneration, antibacterial and antiviral properties, wound healing, and anti-inflammatory and anti-itch effects [9,10].

Currently, the main therapeutic targets for osteoarthritis treatment focus on modulating chondrocyte autophagy, growth factor signaling pathways (e.g., TGF-β/MMP-13), inflammatory responses, and harmful molecular signaling, with additional strategies targeting autophagy inhibition and reactive oxygen species (ROS) reduction [11]. The destabilization of the medial meniscus mouse model, induced by surgical transection of the medial meniscotibial ligament, is widely utilized to mimic post-traumatic OA and evaluate therapeutic interventions [12]. This model is characterized by mild cartilage lesions at 2 weeks post-surgery, followed by rapid subchondral bone lesions, sclerosis, and osteophyte formation [13], while studies have demonstrated its utility in exploring OA-related mechanisms such as autophagy dysregulation, ROS accumulation, and growth factor signaling imbalances [14,15]. The knee joint anatomy across terrestrial mammals is conserved, including bone morphology, ligament/meniscus organization, and cartilage histology [16]. Furthermore, canine orthopedic models have generated significant ethical debates [17]. Meanwhile, the DMM-induced pathologies, including cartilage degeneration, synovial hyperplasia, bone sclerosis, and osteophyte formation, closely parallel naturally occurring OA in canines, which is diagnosable via biomarkers [18,19]. Building on these interspecies parallels, this study evaluates the analgesic and anti-inflammatory efficacy of compound C3GC (800 mg/kgbw/day in mice; 112.5 mg/kgbw/day in dogs) using both DMM-induced OA in mice and naturally occurring OA in retired police dogs. The dosing regimen for the active components was established through a systematic review of the existing clinical and preclinical literature [20,21].

## 2. Materials and Methods

### 2.1. Experimental Products

C3 curcumin glucosamine-chondroitin was sourced from Shenzhen Redray Biotechnology Co., Ltd. (Shenzhen, China), containing 10% Curcumin C3 Complex (from Sabinsa Corporation, Nanjing, China), 20% glucosamine hydrochloride, and 10% chondroitin sulfate. In addition, it also contains dimethyl sulfoxide (DMSO), sodium hyaluronate, undenatured type II collagen, manganese, vitamin E, and other excipients.

### 2.2. Induction of Osteoarthritis in Mice and Treatment

C57BL/6 mice (male, 8–10 weeks old) were obtained from the Animal Center of Huazhong Agricultural University (ID Number: HZAUMO-2023-0328) and were allowed to acclimate with free access to water and food, starting the experiment at 3 months of age. Except for the sham surgery group, all groups underwent arthritis modeling using the destabilization of the medial meniscus (DMM) method. The mice were anesthetized with isoflurane inhalation, initially exposed to 4% isoflurane for 2–3 min via an induction chamber, followed by maintenance anesthesia at 1–2% isoflurane delivered through a face mask with an oxygen flow rate of 1 L/min. The modeling was performed on the right hind limb of the mice, with an incision made near the patellar ligament to open the joint capsule, exposing the medial meniscus ligament, which was then severed to destabilize the meniscus. In the sham surgery group (SHAM), the joint capsule was also opened, and the ligament was visible, but no transverse cutting was performed. In the post-operative phase, the mice received a heated recovery pad aimed at offering warmth and relaxation and were monitored closely until the regaining of ambulation, an indicator of a return to a state of normal mobility. Moreover, all of the animals received a single dose of antibiotics post-surgery.

After one week of recovery, a total of 24 male mice were randomly divided into a SHAM group, a DMM group, a C3GC group, and a GC group, with similar initial body weights. In the C3GC group, C3 curcumin glucosamine-chondroitin was added to the diet at a ratio of 0.5% (0.08% Curcumin + 0.42% other components). In comparison, the GC group was fed a diet containing 0.42% glucosamine-chondroitin and other main components. The SHAM and DMM groups were fed standard feed without any nutritional additives. All of the experimental diets were prepared by Wuhan Wanqian Jiaxing Biotechnology Co., Ltd. (Wuhan, China), using a standardized mixing protocol to ensure homogeneity. The basic components of mouse feed are shown in Supplementary Table S1. Fresh feed was replaced every 72 h. Daily feed intake was recorded per cage, and the average curcumin consumption was estimated as 128 mg/kgbw/day based on a mean daily intake of 4.0 g feed per mouse (body weight 25–30 g). The mice were maintained for 8 weeks post-surgery.

### 2.3. Behavioral Tests Related to Osteoarthritis

The rotarod test apparatus was provided by Anhui Zhenghua Biologic Apparatus Facilities (Huaibei, Anhui, China). The rotarod induces forced exercise to assess functional parameters by measuring the time or endurance of cycling. Each animal is trained twice within 48 h prior to the experiment. Each animal undergoes three rotarod tests, with a 30 min interval, accelerating from 4 rotations per minute to 40 rotations per minute, maintaining the test for a total of 3 min. Each test ends when the animal falls off the rotarod. The time taken for the animal to fall off the rotarod is recorded and used in subsequent analyses, with each animal repeating the test three times.

The time taken for the animal to fall from the top of the rod is measured through the pole test, allowing for the assessment of the animal’s grip strength and agility. The duration of the descent is recorded during the experiment. During the test, the mice were placed with their heads facing upwards on top of the rod, and the animals would typically orient themselves downwards and descend continuously along the rod to return to their housing cage. We recorded the latency period of the animals’ automatic turning (Tturn) and the total time taken to descend to the bottom of the rod (Ttotal), with each animal being tested three times.

### 2.4. Von Frey Test

Mechanical pain was assessed in all mice using an electronic von Frey aesthesiometer (ZS-CITY Beijing Zhongshi-Dichuang Science and Technology Development Co., Ltd., Beijing, China). The instrument was configured with a probe for mice, and the mice were acclimated in a single chamber on top of a metal wire mesh platform for 10 min prior to the test. The von Frey filaments were used to stimulate the plantar surface of the hind paw with increasing force to determine tactile sensitivity. A positive response is indicated by the hind paw withdrawal reflex when stimulation is applied, and the instrument analyzes and records the maximum force applied during stimulation. After each mechanical pain measurement, the mice were allowed to rest for 5 min, and each animal was tested five times.

### 2.5. Micro-CT

At Week 8, after anesthetizing and euthanizing the mice, blood was collected and the right knee joint was immediately harvested, including part of the femur and tibia, with skin and excess tissue removed. After fixation in 4% formaldehyde for 24 h, four samples were randomly selected from each group and scanned using a micro-computed tomography scanner (Skyscan 1276 Micro-CT, manufactured by Bruker Magnetic Resonance, Ettlingen, Germany). The area below the joint surface of the tibial plateau was obtained for analysis. The parameters included trabecular separation (Tb.Sp, mm) and bone mineral density (BMD).

### 2.6. Histological Evaluation of Mouse Joint Tissues

After euthanizing the mice, the affected limbs’ joints from each group were collected. The cartilage tissues from each group were fixed in 4% formaldehyde for 24 h and decalcified with 5% formic acid for 1 week. Subsequently, the cartilage from each group was dehydrated, embedded in paraffin, and sectioned into 5 μm tissue slices. The degeneration of each knee joint’s articular cartilage was assessed using Safranin O-fast green staining and hematoxylin–eosin staining. The degenerative changes were evaluated and analyzed using the Osteoarthritis Research Society International (OARSI) scoring system [22,23].

### 2.7. Assessment of Antioxidant and Anti-Inflammatory Effects

The oxidative stress levels in serum were assessed using a superoxide dismutase (SOD) typing test kit (hydroxylamine method), and the serum inflammatory factor levels were evaluated using a mouse tumor necrosis factor-alpha (TNF-α) enzyme-linked immunosorbent assay kit. The kits were sourced from NanJing JianCheng Bioengineering Institute (Nanjing, China).

Complete blood counts were performed before the experiment began and at the time of euthanasia, measuring the total white blood cell count (WBC), lymphocyte count (LYM), red blood cell count (RBC), and hematocrit (HCT). Additionally, biochemical blood tests were conducted to measure serum creatinine (Crea), blood urea nitrogen (BUN), alanine aminotransferase (ALT), aspartate aminotransferase (AST), and alkaline phosphatase (ALP). Three samples from each group were randomly selected for testing. The results are included in the Supplementary Materials (Tables S2 and S3).

### 2.8. Study of the Anti-Inflammatory and Analgesic Effects of C3GC on Dogs with Osteoarthritis

A cohort of 12 retired police dogs (8 males, 4 females; age 6–9 years; body weight 19.64 ± 6.811 kg) with osteoarthritis was randomly selected from a Police Dog Facility in Nanjing, China. All of the dogs were evaluated by a veterinarian and diagnosed with joint pain. The canine cohort comprised three distinct working breeds, as follows: Springer Spaniels (*n* = 6), German Shepherds (*n* = 5), and Chinese Kunmin (*n* = 1). The dogs were randomly assigned to the C3GC treatment group (*n* = 6) or the control group (*n* = 6), ensuring equivalent distributions of body weight, gender, and breed. The specific statistics of the experimental dogs after grouping are shown in Supplementary Table S4. Inflammatory marker levels and levels of pain were assessed by blind observers. Throughout the study period, all dogs received a standardized maintenance diet provided by Nanjing Police Canine Nutrition Center (Nanjing, China). The feed formula is presented in Supplementary Table S5. C3GC was administered at a daily dose of 112.5 mg/kgbw added to the diet of the dogs. The trial lasted for one month, during which the base’s caretakers administered oral tablets to the experimental dogs. A modified subjective pain score test (referenced HCPI, Supplementary Figure S1) and serum MMP3 and TNF-α testing were conducted at the beginning and end of the trial. The MMP3 kit was sourced from Jiangsu Enzyme Immuno Industrial Co., Ltd. (Yancheng, Jiangsu, China).

### 2.9. Statistical Analysis

The data in this experiment are presented as the mean ± standard deviation (Mean ± SD). Statistical analyses were performed with GraphPad Prism 9.0 (GraphPad Software, San Diego, CA, USA). For multigroup comparisons, one-way ANOVA was used to assess the significance of intergroup parameters. Comparisons between the two groups were conducted using an unpaired *t*-test. *p* < 0.05 is considered statistically significant, and *p* < 0.01 is considered highly significant.

The dog trial data were analyzed using a two-way ANOVA for within-group comparisons. Prior to the analysis, Grubbs’ test was conducted to identify and exclude outlier data.

## 3. Results

### 3.1. C3GC Improves Motor Functions and Helps in Restoring Functions in OA Mice

**Figure 1:**
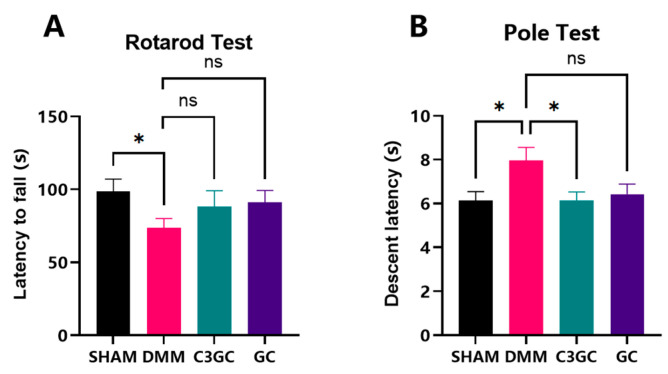
C3GC supplementation mitigates motor impairment in osteoarthritic mice at eight weeks post-surgery. (**A**) Latency-to-fall during rotarod testing quantifies forced exercise endurance in mice. The DMM group showed a significant reduction in motor tolerance (*p* < 0.05 vs. SHAM), while C3GC-treated mice showed a degree of restoration, but without statistical significance (*p* = 0.2493 vs. DMM). (**B**) Pole test descent latency evaluates murine motor function and movement efficiency. C3GC improved latency in descending in comparison with the DMM group (*p* < 0.05), implying an improvement in motor coordination. Data presented as Mean ± SD, *n* = 6, * *p* < 0.05, ns = not significant.

The experiment tested the motor coordination function of mice to examine the effects of C3GC on the symptoms of osteoarthritis and the behavior of affected animals. In the rotarod test, the duration of rotarod performance in DMM mice was significantly decreased compared to that of the SHAM group (*p* < 0.05), while the results for the mice fed C3GC and GC showed a statistically insignificant increase compared to those of the DMM group. In the pole test, there was a significant difference between the SHAM group and the DMM group (*p* < 0.05) in the eighth week, indicating a certain decline in the motor ability of the animals after surgery. Meanwhile, the completion time of the surgical mice fed with C3GC was significantly better than that of the surgical mice not fed with C3GC, thus demonstrating that C3GC has a notable enhancement effect on the motor ability of osteoarthritic mice (Figure 1).

### 3.2. C3GC Alleviates Mechano-Hyperalgesia in Osteoarthritis

**Figure 2:**
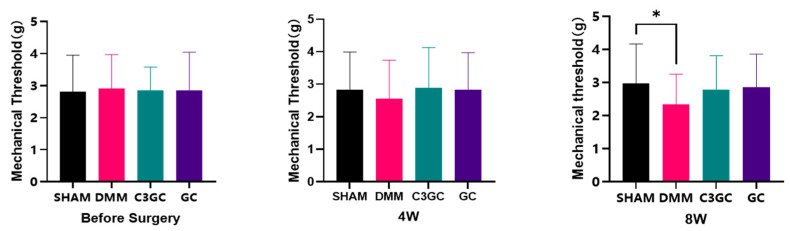
C3GC regulates the mechanosensitivity in osteoarthritic mice. The DMM group showed a significant reduction in the threshold of withdrawal (*p* < 0.05 vs. SHAM), suggesting enhanced pain sensitivity. C3GC supplementation partially restored thresholds, but the differences were not statistically significant (*p* > 0.05 vs. DMM). Data are presented in Mean ± SD, *n* = 6, * *p* < 0.05.

Von Frey tests were conducted to quantify the mechanical sensitivity observed in mice after the experiment. There were no significant differences between the groups prior to the experiment. At Week 8, the mechanical pain threshold of the DMM mice was significantly lower compared to that of the SHAM group, while the C3GC group and the GC group showed mean increases of 18.58% and 21.56%, respectively, compared to the DMM group (Figure 2).

### 3.3. C3GC Protects Subchondral Bone Integrity and Prevents Degenerating Cartilage in Osteoarthritis

#### 3.3.1. Subchondral Bone Remodeling and Degeneration Association

**Figure 3:**
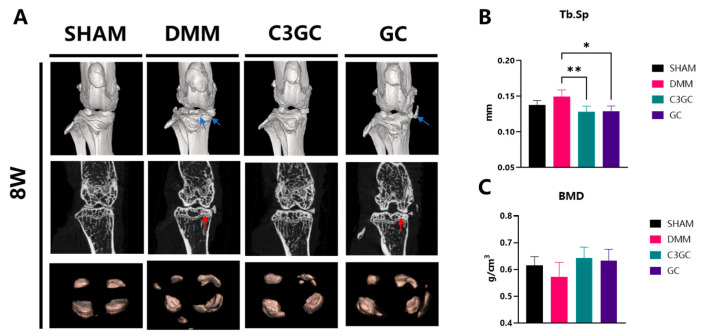
Micro-CT images of the affected limb joints of mice and ROI area analysis results at eight weeks post-surgery. (**A**) Micro-CT 3D reconstruction or 2D representative images of each group, with blue arrows indicating meniscus calcification and joint bone spurs and red arrows indicating bone sclerosis of the medial plateau of the tibia. The increased mineralized tissue was visualized in 3D (distal view). (**B**,**C**) Analysis of trabecular separation (Tb.Sp) and bone mineral density (BMD) in the ROI area of mouse samples at the eighth week after feeding through micro-CT. All data are presented as Mean ± SD, *n* = 4, * *p* < 0.05, ** *p* < 0.01.

Surgical joint samples were collected from mice with post-operative osteoarthritis to investigate the effects of C3GC on bone spur formation and osteoporosis. Three-dimensional visualization revealed calcified tissue manifestations, including meniscal enlargement and ectopic mineralization nodules of various sizes. At eight weeks of treatment, bone spurs or meniscal calcification developed on the surgical joint surfaces. The mice treated with C3GC showed a significant reduction in Tb.Sp in the tibial plateau region (*p* < 0.01), indicating altered bone structure, while a notable reduction (*p* < 0.05) was observed in the GC-treated group, as compared to the DMM group of mice. Mice that were simultaneously administered C3GC and chondroitin showed an increase in BMD, but there was no significant difference compared to the DMM group (Figure 3).

#### 3.3.2. C3GC Preserves Cartilage and Reduces the Severity of Osteoarthritis

**Figure 4:**
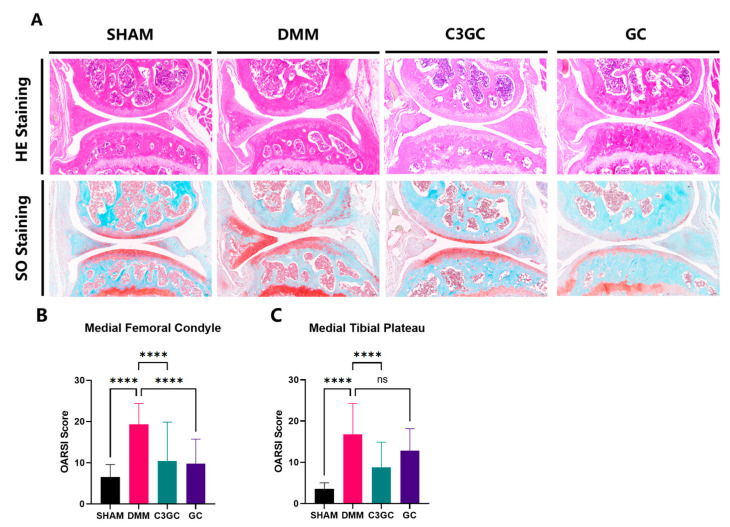
C3GC protects cartilage integrity and attenuates osteoarthritic degeneration in mice. (**A**) Representative SO- and HE-stained sections revealing cartilage integrity (4×). DMM mice revealed severe surface fibrillation, subchondral bone remodeling, and proteoglycan depletion, whereas C3GC-treated mice showed a preserved cartilage structure with reduced matrix degradation. (**B**,**C**) The OARSI score of the medial femoral condyle and medial tibial plateau revealed a significant cartilage loss in DMM mice (*p* < 0.0001 vs. SHAM). C3GC reduced the OARSI score substantially (*p* < 0.0001 vs. DMM), proposing chondroprotective benefits over treatment with glucocorticoids alone. Data are presented as Mean ± SD, *n* = 4, **** *p* < 0.0001, ns = non-significance.

The OARSI score was implemented through S-O staining and HE staining, and the structural integrity of the knee joint cartilage in mice was examined under a microscope after 8 weeks of feeding. The results showed that the DMM group exhibited severe erosion of the cartilage layer and bone remodeling, while the C3GC group and the GC group only showed a mild loss of surface lamina or erosion to the calcified cartilage. The OARSI score results showed significant osteoarthritis in the medial femoral condyle of the operated mice (*p* < 0.0001). However, the degree of osteoarthritis in the mice treated with C3GC and GC decreased significantly compared to the DMM mice (*p* < 0.0001). Significant osteoarthritis changes were also observed in the medial tibial plateau of the operated mice (*p* < 0.0001), while the mice in the C3GC group showed significant improvement (*p* < 0.0001). There was no significant difference in the GC group when compared to DMM mice (Figure 4).

### 3.4. Curcumin Exerts Anti-Inflammatory Effects in OA Mice Models

**Figure 5:**
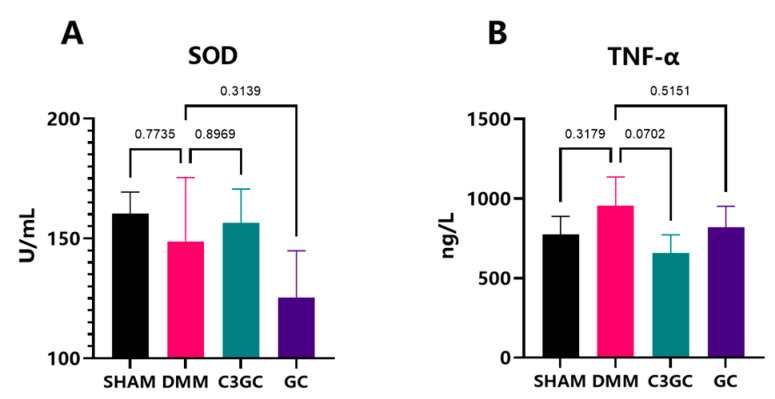
C3GC alleviates systemic inflammation in osteoarthritis. (**A**,**B**) C3GC reduced serum TNF-α levels (*p* = 0.0702 vs. DMM), revealing anti-inflammatory effects. SOD showed an upward trend, suggesting potential oxidative stress modulation (*p* > 0.05). Data are presented as Mean ± SD, *p* value displayed on comparison lines, *n* = 3.

To examine the effects of oral C3GC on serum anti-inflammatory and antioxidant factors, SOD and TNF-α levels were estimated in the serum of mice through ELISA. By Week 8, C3GC was able to decrease the level of TNF-α by 31.4% (*p* = 0.0702), while GC only decreased by 14.3% (*p* = 0.5151). The serum level of SOD in C3GC revealed an increasing trend. The SOD level in the C3GC group exhibited an increase of 8.16% when compared to the DMM group (*p* > 0.05) and a rise of 28.44% in comparison to the GC group (Figure 5).

### 3.5. The Analgesic and Anti-Inflammatory Effects of C3 Curcumin in Dogs with Osteoarthritis

**Figure 6:**
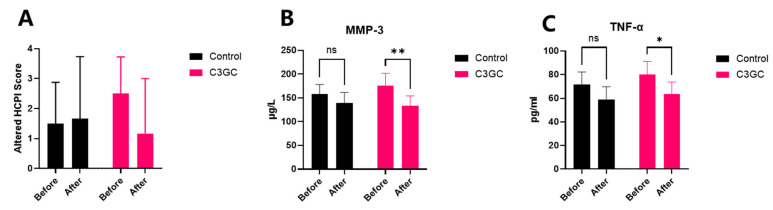
Supplementation with C3GC relieves osteoarthritic dogs from pain and inflammation. (**A**) Subjective pain scores decreased in C3GC-treated dogs on Day 30. (**B**) Serum MMP-3 was notably lower in the C3GC group on Day 30 (*p* < 0.01). (**C**) Serum TNF-α was notably lower in the C3GC group on Day 30 (*p* < 0.05), suggesting chondroprotective and anti-inflammatory effects. Values are expressed as Mean ± SD, * *p* < 0.05, ** *p* < 0.01, ns = non-significance.

Subjective pain scoring and serum inflammatory factor testing were conducted on recruited dogs before (Day 0) and after (Day 30) the 1-month dietary intervention. The subjective pain score decreased by 53.3% after one month of C3GC feeding, although there was no significant difference. In addition to this, the serum levels of MMP-3 significantly dropped by 24.5% compared to pre-treatment (*p* < 0.01), and the TNF-α levels also significantly decreased by 20.8% (*p* < 0.05). No significant changes were observed in the control group between pre-treatment and post-treatment measurements for either subjective pain scores or serum inflammatory markers (Figure 6).

## 4. Discussion

This study demonstrates the role of C3GC in delaying the progression of osteoarthritis in mice and dogs, supported by histopathological, behavioral, and anti-inflammatory evidence. Curcumin is an antioxidant that alleviates pain in osteoarthritis patients and promotes function [6,24]. The combined use of glucosamine and chondroitin exhibits complementary anti-catabolic and anti-inflammatory effects, and its therapeutic efficacy is comparable to that of celecoxib [25]. Other studies have found that chondroitin can alleviate pain symptoms and improve joint function, while glucosamine significantly improves joint stiffness [26]. This study suggests that the use of supplements based on glucosamine and chondroitin can delay cartilage degeneration, preserve the microstructure of subchondral trabecular bone, and promote motor coordination. The incorporation of curcumin leads to improved outcomes, including improved climbing performance, reduced OARSI scores, and decreased levels of TNF-α. Other components in the formulation, such as DMSO and undenatured type II collagen, may provide complementary support. DMSO has been reported to reduce synovial inflammation in OA models [27], while undenatured collagen supplementation was associated with improved mobility in canine osteoarthritis without adverse effects [28].

The symptoms of osteoarthritis in mice involve a complex interaction between pain and functional impairment. Motor functional impairment and indirect pain can be assessed through spontaneous activities (e.g., gait analysis, open-field exploration), whereas forced exercise paradigms, such as rotarod testing, are used to quantify pain-related movement avoidance, endurance, and motor learning [29]. This experiment assessed motor abilities through rotarod testing and found that mice in the C3GC and GC groups exhibited longer latency to fall, which suggested a potential trend of improved motor performance compared to that of the DMM group. However, these differences did not reach statistical significance. The pole test, a validated method for evaluating motor retardation [30], further corroborated the functional decline in DMM mice. It has been widely used in Parkinson’s models because it can evaluate the animal’s motor coordination [31]. We analyzed the flexibility and coordination of animals through the pole test, finding that the DMM group exhibited a significant decline in rod climbing ability, and that C3GC had a significant positive effect on the motor coordination and flexibility of osteoarthritic mice, outperforming the GC group. Pain assessment in animal models of osteoarthritis can be conducted through behavioral experiments [32], and the experimental results verified that C3GC could alleviate functional impairment, suggesting an indirect capacity to mitigate pain caused by osteoarthritis. Additionally, some studies have found that curcumin oil can reverse the prolonged rod climbing time in a Parkinson’s disease mouse model [33].

The von Frey test is commonly used to assess pain in osteoarthritic mice. In traumatic osteoarthritis, the occurrence of inflammation-induced mechanical sensitization leads to a decrease in the mechanical stimulation threshold in animals for both the affected joint and remote areas [34,35]. In the experiment, mechanical pain assessments were conducted on mice, revealing that the pain hypersensitivity threshold decreased after surgery. However, there was no significant difference in mechanical pain responses between the surgical mice treated with C3GC and the sham surgery group, indicating that the addition is beneficial for alleviating osteoarthritis pain. Research has found that topical curcumin nanoparticles can improve mechanical pain performance and alleviate OA-related pain, however, the analgesic effect is not significant when taken orally [5]. Therefore, the control effect of curcumin on osteoarthritis symptoms may be related to the formulation. Curcumin treatment in chikungunya-infected mice can increase their mechanical pain threshold levels [36]. Additionally, studies in rats have found that lipid-particle-loaded curcumin (30 mg/kg) has a protective effect against joint pain hypersensitivity comparable to naproxen (25 mg/kg) [37].

Subchondral bone sclerosis and progressive cartilage degeneration are widely regarded as hallmarks of OA. During this process, abnormal bone remodeling leads to insufficient subchondral bone mineralization, while pathological changes such as microdamage, bone marrow edema-like lesions, and bone cysts occur in the subchondral bone [38]. Therefore, we studied the microstructure of subchondral trabecular bone using micro-CT. We found that, after 8 weeks of OA induction, Tb.Sp increased and BMD decreased, indicating trabecular damage and a decline in bone mineral density. These findings suggest that supplementation with C3GC and GC preserved the microstructure of subchondral trabecular bone in osteoarthritic mice and prevented bone loss. Compared to the GC group, the differences between the C3GC group and the DMM group were more significant, possibly indicating that C3GC had a better therapeutic effect on osteoarthritis. Similar results were observed in mice with knee osteoarthritis. In the fourth week of the experiment, the Tb.Sp level of DMM mice injected intraperitoneally with curcumin was significantly higher than that of the surgery group [39]. The micro-CT results showed that curcumin treatment significantly reduced Tb.Sp and inhibited structural changes in the skeleton of rats that had developed osteoporosis [40].

The pathological features of osteoarthritis include the loss of proteoglycan staining, cartilage fibrosis, and degeneration, damage, and erosion of the cartilage matrix. In our histological analysis, Safranin O-fast green staining was used to assess the extent of cartilage damage in mice. The OARSI semi-quantitative scoring system [22,23] was used to evaluate the histological changes in the knee joints of osteoarthritic mice. In our results, the scores of the DMM group mice significantly increased, demonstrating that osteoarthritis changes occurred in the mice after surgery. Feeding C3GC and GC to mice resulted in a decrease in the OARSI score related to the affected cartilage layer of the joints, especially in the mice that received C3GC. The experimental results indicate that C3GC protects cartilage and slows down cartilage degeneration in osteoarthritis model mice, with effects superior to those of GC. Other studies have demonstrated that both the oral administration of curcumin [5] and the injection of curcumenol [41] can significantly reduce OARSI scores in DMM-induced osteoarthritic mice. Additionally, there is a wealth of literature confirming that chondroitin sulfate and glucosamine exhibit protective effects on cartilage in experimental animal models of osteoarthritis and in companion animals [42]. In osteoarthritis progression, initial cartilage surface erosion is subsequently exacerbated by progressive matrix degradation [43]. These results indicate that C3GC mitigates osteoarthritis through cartilage tissue preservation.

The inflammatory response and oxidative stress in chondrocytes and other joint tissues are associated with the progression and severity of osteoarthritis, making them ideal targets for treatment. Polyphenolic compounds such as curcumin exert their anti-inflammatory and antioxidant activities in osteoarthritis models, inhibiting the inflammatory response and ROS generation during the OA process [44]. The pro-inflammatory factors IL-1β and TNF-α are elevated in the synovial fluid, cartilage, synovium, and subchondral bone of osteoarthritic joints [44]. In this study, the serum levels of TNF-α were elevated in surgically operated mice and decreased after feeding with C3GC, with a greater reduction than that observed in the GC group. In a mouse model of traumatic osteoarthritis, both curcumin and curcumin-encapsulated nanoparticles inhibited the mRNA expression of pro-inflammatory factors such as IL-1β and TNF-α [5]. A study by Wang et al. [40] demonstrated that curcumin can inhibit IL-1β, IL-6, and TNF-α in surgical OA models. They also showed that curcumin alleviates osteoarthritis through the p38MAPK pathway. Superoxide dismutase (SOD) is an important antioxidant enzyme. In this experiment, there is an upward trend in the SOD levels within the C3GC group in comparison to the DMM group and the GC group. In other animal models, formulations containing curcumin have been shown to enhance the levels of SOD, CAT, and GPX, playing a regulatory role in antioxidant enzymes during the progression of OA [45]. A study using curcumin and chondroitin sulfate in rats showed that both the individual use and combined administration of the two can increase serum SOD levels [46].

TNF-α and IL-1β can regulate the expression of MMP-3 in OA through the NF-κb, MAPK, and PI3K/Akt pathways. During the pathogenesis of OA, increased mechanical load and degradation of the cartilage matrix lead to elevated levels of MMP-3, which can promote angiogenesis in the cartilage, the accumulation of inflammatory cells, and the differentiation of mesenchymal cells into chondrocytes [47]. This study detected the levels of MMP-3 and TNF-α in canine serum and found that C3GC can effectively reduce the levels of MMP-3 and TNF-α in osteoarthritic animals, alleviating their pain. Research on OA rats showed that curcumin could significantly downregulate the levels of MMP-3 in their serum [45]. A nutritional supplement obtained by co-micronizing palmitoyl amino glucosamine with curcumin can also reduce the levels of serum MMP-3 and inflammatory factors TNF-a and IL-1β induced by sodium iodoacetate [48]. These studies indicate that curcumin and its extract formulations can improve the inflammatory response in osteoarthritis to varying degrees.

This study has certain limitations. To mitigate these issues, several methodological refinements were implemented to address potential confounding factors. In the murine studies, daily supplement intake was quantified via feed weight monitoring to compensate for dosing uncertainties, as C3GC was incorporated into the feed under ad libitum conditions. By comparing the C3GC group with the GC control group, the improved effects specifically attributed to the incorporation of C3 in the C3GC composition were highlighted. In the canine trials, although lacking a placebo group, single-blinded assessment protocols coupled with serum inflammatory marker quantification provided objective efficacy evaluation.

## 5. Conclusions

This study investigated the effect of C3GC by using murine behavior tests, stifle micro-CT, cartilage pathological section, cytokines levels, and subjective pain scores in canines. The treatment with C3GC and GC—especially C3GC—can relieve pain and symptoms, preserve cartilage, and protect the microstructure of the subchondral trabecular bone in the DMM surgical mouse model. C3GC can also significantly reduce levels of MMP-3 and TNF-α in dogs with osteoarthritis. Moreover, further research is needed to identify the optimal biological effect dosage and establish a foundation for clinical translation and application.

## Data Availability

The data presented in this study are available upon request from the corresponding author.

## Supplementary Materials

The following supporting information can be downloaded at: https://www.mdpi.com/article/10.3390/vetsci12050462/s1, Table S1: Nutritional components of mouse food; Table S2: Biochemical results of mice post-feeding; Table S3: CBC results of mice after feeding; Table S4: Statistical data of experimental dogs after grouping; Table S5: Nutritional components of dog food; Figure S1: Altered HCPI scoring.
