# Supplementary material for: A Nutritional Supplement Containing Curcumin C3 Complex, Glucosamine, and Chondroitin Alleviates Osteoarthritis in Mice and Canines
Source: Vet Sci. 2025 May 12;12(5):462. doi: 10.3390/vetsci12050462 (PMC12115434; doi:10.3390/vetsci12050462)
Supplement: Supplementary file 1 [file vetsci-12-00462-s001.zip › vetsci-3528674-supplementary.pdf]

Table S1. Nutritional components of mice food.

Content	%
Crude protein	18
Crude fat	4
Water	10
Crude fiber	5
Ash content	8
Ca	1.0-1.8
P	0.6-1.2
Lysine	8.2
DL-Methionine	0.53

Table S2. Biochemical results of mice post-feeding. Data are presented as Mean±SD, n=3/ group.

	ALT U/L	AST U/L	BUN mmol/L	ALP U/L	Crea mmol/L
Reference range	22-133	46-221	2–71	16-200	9-159
SHAM Group	32.57±7.23	46.5±10.69	10.5±1.76	73.03±20.21	23.67±6.03
DMM Group	27.5±0.35	45.8±4.65	9.76±1.55	51.97±3.26	29.67±5.51
C3GC Group	32.33±4.74	47.73±3.69	11.31±1.45	65.93±5.77	27.67±3.06
GC group	20.43±1.89	81.83±63.45	11.08±0.82	63.83±6.27	25±1

Table S3. CBC results of mice. Data are presented as Mean ± SD, n=3/ group.

	WBC 10^9/L	LYM 10^9/L	MON 10^9/L	NEU 10^9/L	RBC 10^{12}/L	HGB g/L	HCT %	MCH Pg	PLT 10^9/L
Reference Range	6.00-15.00	3.40-7.44	0.00-0.6	0.50-3.80	7.00-12.00	100-190	35.00-45.00	21.1-25.2	200-450
SHAM	6.35±6.71	3.5±2.8	0.14±0.18	3.38±5.35	7.46±3.19	9.38±4.58	31.29±12.53	12.33±1.09	340.75±175.35
DMM	5.37±4.12	4.68±3.76	0.15±0.13	0.55±0.28	9.11±2.51	12.3±3.58	38.64±10.49	13.42±0.35	404.8±256.6
C3GC	2.95±1.02	2.42±1.04	0.12±0.08	0.42±0.18	8.75±0.53	11.5±0.7	37.36±2.24	13.15±0.31	229±195.43
GC	3.58±1.25	3.09±1.07	0.11±0.07	0.38±0.15	9.89±0.31	13.27±0.61	43.07±1.68	13.4±0.2	636.33±17.47

Table S4. Statistical data of experimental dogs after grouping

Animal information		Control	C3GC
Gender	Male	4	4
	Female	2	2
Breeds	Chinese Kunmin	1	0
	German shepherd	2	3
	Springer Spaniel	3	3
Body Weight (kg)	Before	20.87±8.678	18.42±4.812
	After	19.62±7.41	18.68±4.976
BCS	Before	4.5±0.8367	5.167±1.941
	After	4.167±0.7528	4.833±0.9832

Table S5. Nutritional components of dog food

Content	%
Protein	26
Crude fat	12
Water	8
Crude fiber	4.2
Ash content	9.3
Ca	1.1
P	0.8
Cl^-	0.24
Lysine	1.1

Figure S1. Altered HCPI Scoring

Altered HCPI

1.Rate your dog's attitude and/or mood:

0	1	2	3	4
Very alert	Alert	Neither alertnordisinterested	Disinterested	Verydisinterested/lethargic

2.Rate your dog's willingness to participate in play or interact:

0	1	2	3	4
Very willing	Willing	Reluctant	Very reluctant	Does not participate or interact at all

3.Rate your dog's eagerness to walk:

0	1	2	3	4
Very eager	Eager	Reluctant	Very reluctant	Does not wantto walk at all
